# Neuropilin 1 (NRP1) Positively Regulates Adipogenic Differentiation in C3H10T1/2 Cells

**DOI:** 10.3390/ijms24087394

**Published:** 2023-04-17

**Authors:** Yaqiong Yu, Yoko Uchida-Fukuhara, Yao Weng, Yuhan He, Mika Ikegame, Ziyi Wang, Kaya Yoshida, Hirohiko Okamura, Lihong Qiu

**Affiliations:** 1 Department of Endodontics, School and Hospital of Stomatology, China Medical University, Liaoning Provincial Key Laboratory of Oral Diseases, Shenyang 110002, China; 2 Department of Oral Morphology, Graduate School of Medicine, Dentistry and Pharmaceutical Sciences, Okayama University, Okayama 700-8525, Japan; 3 Department of Orthodontics, Graduate School of Medicine, Dentistry and Pharmaceutical Sciences, Okayama University, Okayama 700-8525, Japan; 4 Department of Oral Healthcare Education, Institute of Biomedical Sciences, Tokushima University Graduate School, Tokushima 770-8504, Japan

**Keywords:** neuropilin 1, adipogenic differentiation, mesenchymal stem cells

## Abstract

Neuropilin 1 (NRP1), a non-tyrosine kinase receptor for several ligands, is highly expressed in many kinds of mesenchymal stem cells (MSCs), but its function is poorly understood. In this study, we explored the roles of full-length NRP1 and glycosaminoglycan (GAG)-modifiable NRP1 in adipogenesis in C3H10T1/2 cells. The expression of full-length NRP1 and GAG-modifiable NRP1 increased during adipogenic differentiation in C3H10T1/2 cells. NRP1 knockdown repressed adipogenesis while decreasing the levels of Akt and ERK1/2 phosphorylation. Moreover, the scaffold protein JIP4 was involved in adipogenesis in C3H10T1/2 cells by interacting with NRP1. Furthermore, overexpression of non-GAG-modifiable NRP1 mutant (S612A) greatly promoted adipogenic differentiation, accompanied by upregulation of the phosphorylated Akt and ERK1/2. Taken together, these results indicate that NRP1 is a key regulator that promotes adipogenesis in C3H10T1/2 cells by interacting with JIP4 and activating the Akt and ERK1/2 pathway. Non-GAG-modifiable NRP1 mutant (S612A) accelerates the process of adipogenic differentiation, suggesting that GAG glycosylation is a negative post-translational modification of NRP1 in adipogenic differentiation.

## 1. Introduction

Adipogenesis is a complicated process that involves multipotent stromal cell commitment toward preadipocytes, followed by proliferation and differentiation of preadipocytes into mature adipocytes [1]. The imbalance of adipogenesis leads to disorders of metabolic regulation and energy homeostasis [2]. It is well known that the self-renewal and differentiation of mesenchymal stem cells (MSCs) are tightly regulated by transmembrane receptors, which respond to a wide range of surrounding environments by converting their morphology, behavior, and fate decisions accordingly [3,4].

Neuropilin 1 (NRP1) is a highly conserved transmembrane glycoprotein and plays a co-receptor role for multiple ligands, such as class 3 semaphorins (Sema3A) [5], vascular endothelial growth factor (VEGF) [6], and platelet-derived growth factor (PDGF) [7]. Because NRP1 associates with several different types of receptors, it variably promotes angiogenesis, neural development, cytoskeleton remodeling, initial immune response, and differentiation of stem cells. Recently, NRP1 has also been identified as a novel mediator responsible for the keratinocyte growth factor (KGF)-dependent pathway, promoting adipogenesis of adipose-derived MSCs [8]. NRP1 undergoes post-translational glycosaminoglycan (GAG) glycosylation modifications at Ser612 [6]. The GAG glycosylation of NRP1 at Ser612 plays an important role in the modulation of VEGF signaling, cell proliferation and migration, and cancer invasion [6,9]. However, the role of NRP1 and its GAG-modifiable form in the multidirectional differentiation of MSCs is poorly characterized and understood.

C-Jun N-terminal kinase (JNK)–interacting protein 4 (JIP4, also known as Spag9) was first identified as a scaffold for mitogen-activated protein kinase (MAPK) signaling modules [10]. Subsequently, several studies have shown that JIP4 has a multifunctional role as an adaptor for kinesin and dynein–dynactin complex, and that JIP4 is involved in many cellular processes, including axonal outgrowth, muscle development, endosomal and mitochondrial transport, as well as actin cytoskeleton and membrane trafficking organization [11,12,13,14]. JIP4 is also a direct adaptor for membrane EGF receptors (EGFR), which are master regulators of receptor tyrosine kinase (RTK) autophosphorylation and the channeling of mitogenic signals predominantly via the Ras-extracellular signal-regulated kinase (ERK), Akt, and JNK pathways [11]. Phosphorylated Akt contributes to adipogenic differentiation, whereas inactivation of the Akt pathway inhibits adipogenesis [1]. Moreover, phosphorylated ERK1/2 activation has been shown to be essential for the induction of adipogenesis, as confirmed by the intervention with an ERK 1/2 inhibitor [15]. However, it is still unclear whether JIP4 is expressed in MSCs, and how the interaction of JIP4 with membrane proteins occurs during the adipogenic differentiation of MSCs.

The specific purpose of this study was to investigate the role of NRP1 in the response to the adipogenic induction in C3H10T1/2 cells, and to reveal the underlying molecular mechanisms of full-length NRP1 and its GAG-modifiable form in adipogenic differentiation. We demonstrated that the full-length NRP1 and its GAG-modifiable form were involved in adipogenesis in C3H10T1/2 cells. NRP1 binds to JIP4 and promotes adipogenic differentiation by activating the Akt and ERK1/2 signaling pathways. Interestingly, there is a novel observation that overexpression of non-GAG-modifiable NRP1 mutant (S612A) promotes adipogenic differentiation in C3H10T1/2 cells. These findings elucidated a new mechanism of NRP1-mediated adipogenesis of C3H10T1/2 cells.

## 2. Results

### 2.1. NRP1 Protein Expression Increases and Its Subcellular Localization Slightly Changes during Adipogenesis in C3H10T1/2 Cells

**Figure 1:**
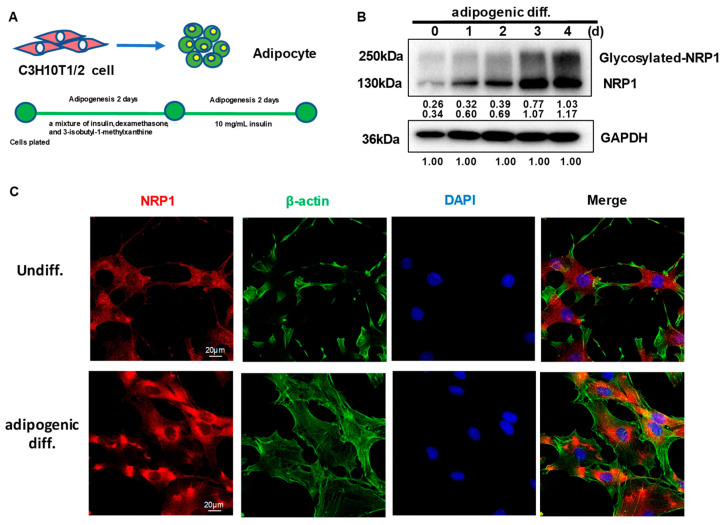
Expression and subcellular localization of NRP1 during adipogenesis in C3H10T1/2 cells. (**A**) C3H10T1/2 cells have the capacity to differentiate into adipocytes. (**B**) C3H10T1/2 cells were cultured in an adipogenic medium for the indicated days; Western blot analysis was performed using anti-NRP1 antibody. (**C**) C3H10T1/2 cells were cultured in an adipogenic medium for three days; immunofluorescence staining and confocal laser scanning microscopy detected NRP1, β-actin, and DAPI. Scale bars, 20 µm. The images are representative of at least three independent experiments.

C3H10T1/2 cells can differentiate into adipocytes (Figure 1A). After the induction of adipogenic differentiation, the expression levels of full-length NRP1 and glycosylated NRP1 protein gradually increased (Figure 1B). During adipogenic differentiation from stem cells, cellular morphological changes occur by remodeling of the actin cytoskeleton. Before differentiation induction, C3H10T1/2 cells exhibited similar fibroblast-like morphology (Figure 1C). After differentiation induction into adipocytes for three days, C3H10T1/2 cells gradually changed to round or polygonal epithelioid cells. In addition, the localization of NRP1 in cytoplasm slightly changed, which was manifested as perinuclear aggregation (Figure 1C). Although NRP1 is a membrane protein, it was distributed in both the cell membrane and cytoplasm in C3H10T1/2 cells (Figure 1C).

### 2.2. Knockdown of NRP1 Represses Adipogenesis of C3H10T1/2 Cells

**Figure 2:**
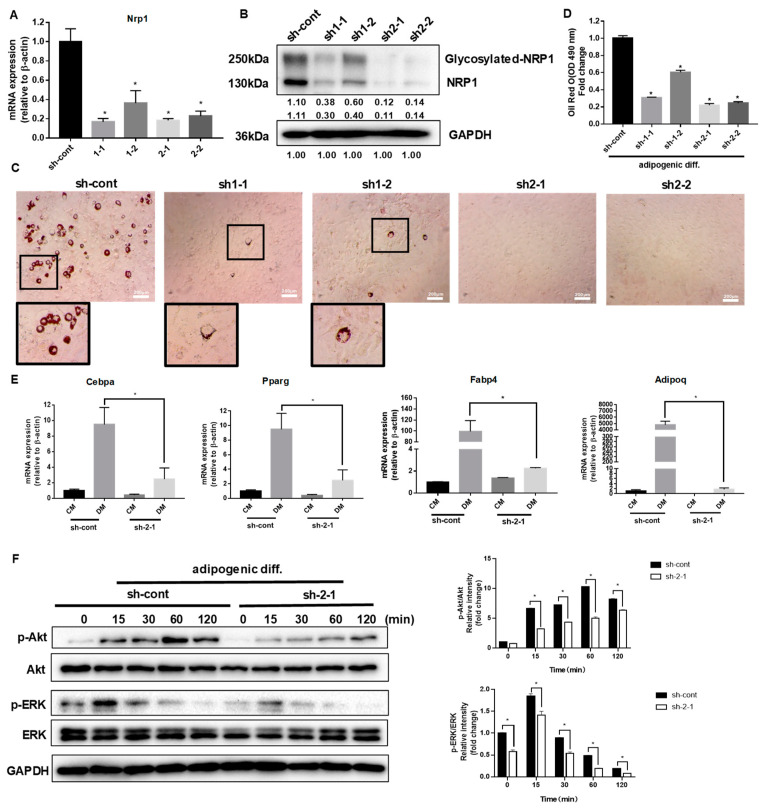
Effect of NRP1 knockdown on adipogenesis in C3H10T1/2 cells. (**A**,**B**) qPCR and Western blot analysis for NRP1 were performed in NRP1-knockdown cells. (**C**) Oil red O staining of NRP1-knockdown cells with adipogenic induction for four days. Scale bars, 200 µm. (**D**) Lipid accumulation was assessed by quantification of oil red O staining. (**E**) qPCR analysis for Cebpa, Pparg, Fabp4, and Adipoq in sh-cont and sh-2-1cells with or without adipogenic induction for four days. (**F**) NRP1-knockdown cells were cultured in an adipogenic medium for the indicated time, and Western blot analysis was performed using anti-Akt, phosphorylated Akt, ERK and phosphorylated ERK antibodies. The images are representative of at least three independent experiments. Control medium (CM); differentiation medium (DM). The data are presented as mean ± standard deviation (SD) (*n* = 3 independent experiments) * *p* < 0.05.

C3H10T1/2 cells were infected with a control lentivirus (sh-cont) or with lentiviruses containing shRNA for NRP1 (sh1 and sh2). The stable single-cell clones (sh1-1, sh1-2, sh2-1, and sh2-2) were selected. Sh2-1 showed the highest decrease in NRP1 mRNA (Figure 2A) and protein expression levels (Figure 2B), while sh1-2 was less effective in reducing NRP1 mRNA (Figure 2A) and protein expression levels than other groups (Figure 2B).

After the induction of adipogenic differentiation, numerous neutral lipid droplets stained with oil red O were observed in C3H10T1/2 cells. NRP1 silencing resulted in a decreased number of neutral lipid droplets as determined by direct observation or quantification (Figure 2C,D). Sh2-1 and sh2-2 even completely abolished the formation of neutral lipid droplets (Figure 2C). The well-known transcription factors controlling adipogenesis are CCAAT/enhancer binding protein (C/EBP) α and peroxisome proliferator-activated receptor (PPAR) γ, which transactivate subsets of adipocyte-specific genes, such as fatty acid-binding protein 4 (FABP4) and adiponectin. The mRNA expression levels of Cebpa, Pparg, Fabp4, and Adipoq decreased to different degrees in Sh2-1 cells, compared with the control group (Figure 2E). The PI3K/Akt and MAPK families signaling pathways, ERK1/2, JNK, and p38 kinase, are important intracellular signal transmitters that are involved in the regulation of adipogenic differentiation. We examined Akt and ERK1/2 protein levels in the early stage of adipogenic differentiation in NRP1-knockdown cells. Knockdown of NRP1 markedly reduced the phosphorylation levels of Akt and ERK1/2 (Figure 2F).

### 2.3. Scaffold Protein JIP4 Silencing Represses Adipogenic Differentiation

**Figure 3:**
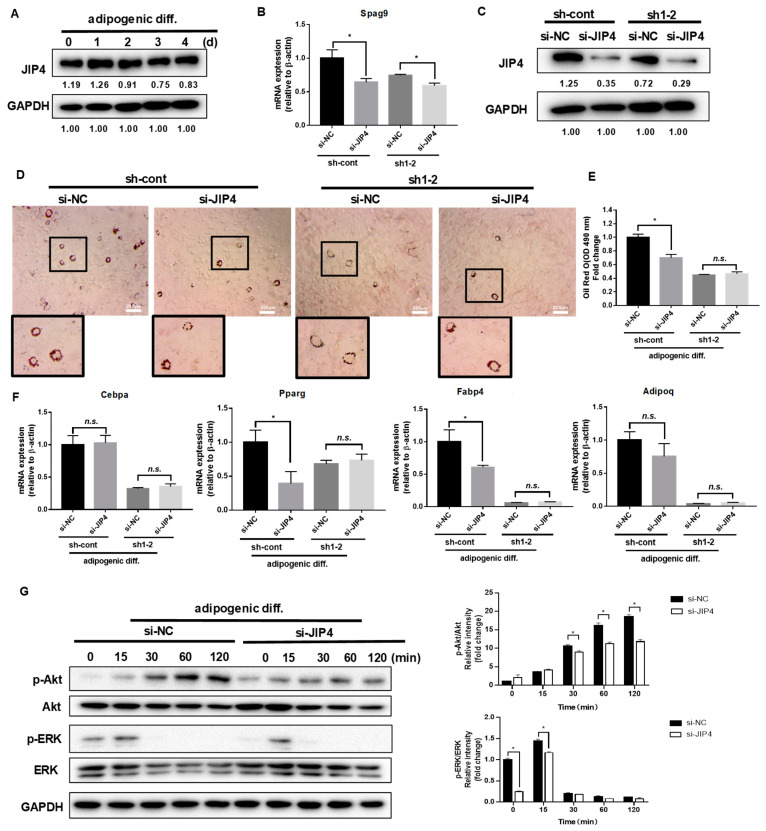
Effect of JIP4 silencing on adipogenesis in C3H10T1/2 cells. (**A**) C3H10T1/2 cells were cultured in an adipogenic medium for the indicated days, and Western blot analysis was performed using anti-JIP4 antibody. (**B**,**C**) qPCR and Western blot analysis for JIP4 were performed in NRP1-JIP4–double-knockdown cells. (**D**) Oil red O staining of NRP1-JIP4–double-knockdown cells with adipogenic induction for four days. Scale bars, 200 µm. (**E**) Lipid accumulation was assessed by quantification of oil red O staining. (**F**) qPCR analysis for Cebpa, Pparg, Fabp4, and Adipoq in NRP1-JIP4–double-knockdown cells with or without adipogenic induction for four days. (**G**) JIP4-knockdown cells were cultured in an adipogenic medium for the indicated time, and Western blot analysis was performed using anti–Akt, phosphorylated Akt, ERK and phosphorylated ERK antibodies. The images are representative of at least three independent experiments. The data are presented as mean ± standard deviation (SD) (*n* = 3 independent experiments) * *p* < 0.05.

In our study, JIP4 remained highly expressed before and after adipogenic differentiation in C3H10T1/2 cells (Figure 3A). To analyze the roles of JIP4 in adipogenesis, JIP4 expression was downregulated using siRNA for JIP4 (siJIP4) and negative control (siNC) in NRP1-knockdown cells (sh-cont and sh1-2). Inhibition of JIP4 was confirmed at both mRNA level (Figure 3B) and protein level (Figure 3C). Si-JIP4 was effective in reducing the accumulation of lipid droplets in sh-cont cells; however, si-JIP4 did not affect the level of lipid accumulation in sh1-2 cells, as visualized by oil red O staining (Figure 3D,E). To study the effect of JIP4 silencing on the expression of adipogenic markers, we carried out qPCR for Cebpa, Pparg, Fabp4, and Adipoq. The mRNA levels of Pparg and Fabp4 markedly decreased after knockdown of JIP4 in sh-cont cells with adipogenic induction for four days (Figure 3F). However, Cebpa, Pparg, Fabp4, and Adipoq remained unaltered after JIP4-silencing in sh1-2 cells (Figure 3F). Hence, we demonstrated that individual knockdown of JIP4 caused inhibition of adipogenic differentiation. However, for inhibition of adipogenic differentiation, double knockdown of NRP1 and JIP4 at the same time did not show significant superiority over individual knockdown of NRP1. Meanwhile, the cells treated with JIP4 siRNA showed greatly reduced phosphorylation levels of Akt and ERK1/2 (Figure 3G).

### 2.4. NRP1 Interacts with JIP4 in Adipogenic Differentiation

**Figure 4:**
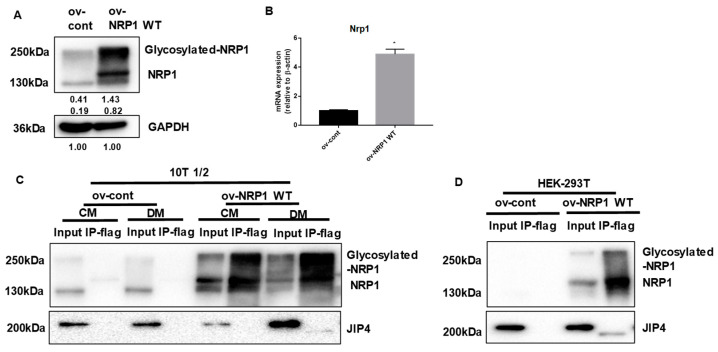
NRP1 binds to JIP4 in adipogenic differentiation. (**A**,**B**) qPCR and Western blot analysis for NRP1 were performed in NRP1 wild-type (WT) flag-overexpressing cells and control cells. Samples from ov-NRP1 WT and ov-cont cells from C3H10T1/2 cells (**C**) or HEK 293T cells (**D**) were immunoprecipitated with anti-Flag antibody–conjugated agarose and subjected to Western blot analysis. Whole-cell lysate from the cells was used as a positive control (input). The blotted membranes were incubated with anti-NRP1 antibody (upper panel) or anti-JIP4 antibody (lower panel). The images are representative of at least three independent experiments. Immunoprecipitation (IP); control medium (CM); differentiation medium (DM). The data are presented as mean ± standard deviation (SD) (*n* = 3 independent experiments) * *p* < 0.05.

We next examined in greater biochemical detail whether NRP1 may promote adipogenic differentiation by binding JIP4. First, NRP1 WT overexpression was achieved through transduction of lentivirus containing the NRP1 gene. The expression levels of NRP1 in these cellular models were determined using qPCR and Western blotting (Figure 4A,B). Second, to assess whether JIP4 binds to NRP1, proteins prepared from ov-NRP1 WT and ov-cont cells were immunoprecipitated with Flag affinity gel and then by Flag peptide elution, and subjected to Western blot analysis. Figure 4C shows that anti-JIP4 antibody interacted with Flag-NRP1 complex eluted from the Flag affinity gel. Interestingly, the molecular weight of the positive band was estimated at 200 kDa in the input group, but it was slightly lower in the immunoprecipitation (IP) group (Figure 4C, lower panel, lane 8). A similar positive band was also detected in the samples prepared from ov-NRP1 WT with Flag of HEK 293T cells in the present system (Figure 4D lower panel, lane 4). Altogether, our results suggest that NRP1 may recruit JIP4 during adipogenic differentiation in C3H10T1/2 cells.

### 2.5. Nonmodifiable NRP1 Mutant (S612A) Promotes Adipogenic Differentiation

**Figure 5:**
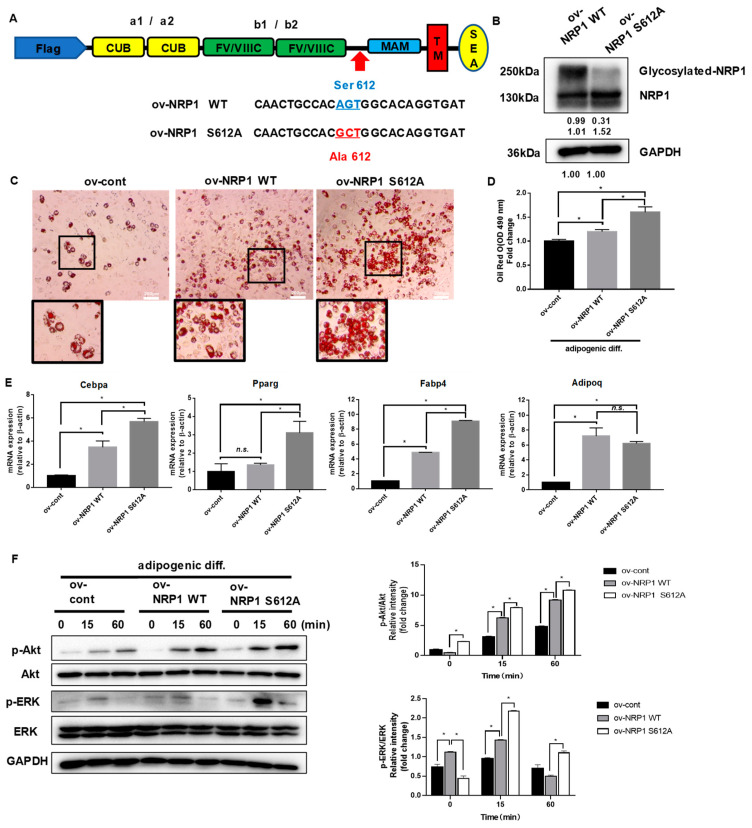
Effects of nonmodifiable NRP1 mutant (S612A) on adipogenic differentiation. (**A**) Design of lentivirus constructs. Ser612 exists in the bridge region between the b1b2 and MAM domains. (**B**) Western blot analysis for NRP1 was performed in ov-NRP1 WT and ov-NRP1 S612A cells. (**C**) Oil red O staining of ov-cont, ov-NRP1 WT, and ov-NRP1 S612A cells with adipogenic induction for four days. Scale bars, 200 µm. (**D**) Lipid accumulation was assessed by quantification of oil red O staining. (**E**) qPCR analysis for Cebpa, Pparg, Fabp4, and Adipoq in ov-cont, ov-NRP1 WT, and ov-NRP1 S612A cells with adipogenic induction for four days. (**F**) Ov-cont, ov-NRP1 WT, and ov- NRP1 S612A cells were cultured in an adipogenic medium for the indicated time, and Western blot analysis was performed using anti–Akt, phosphorylated Akt, ERK, and phosphorylated ERK antibodies. The images are representative of at least three independent experiments. The data are presented as mean ± standard deviation (SD) (*n* = 3 independent experiments) * *p* < 0.05.

It is well established that NRP1 can be post-translationally modified by the addition of GAGs at Ser612 [6]. We further generated an NRP1-encoding lentivirus construct, NRP1 S612A, in which Ser612 was replaced by Ala612 (Figure 5A). The prominent overexpression of NRP1 WT and NRP1 S612A was confirmed by Western blot (Figure 5B). Overexpression of NRP1 S612A promoted adipogenic differentiation in C3H10T1/2 cells more than overexpression of NRP1 WT did, as verified by the increased amount of lipid droplet accumulation in oil red O staining assay (Figure 5C,D). The mRNA levels of Cebpa, Pparg, and Fabp4 were markedly increased in ov-NRP1 S612A cells compared with ov-NRP1 WT cells (Figure 5E). However, the mRNA level of Adipoq did not significantly change (Figure 5E). Next, we investigated the effects of NRP1 S612A overexpression on the signaling pathways known to mediate adipogenic differentiation. Western blot results showed that overexpression of NRP1 S612A also markedly increased the phosphorylation levels of Akt and ERK1/2 compared with overexpression of NRP1 WT (Figure 5F). Taken together, these results show that glycosylation of NRP1 at S612A decelerates adipogenic differentiation, suggesting glycosylation at S612A as a negative post-translational modification of NRP1 in adipogenic differentiation.

## 3. Discussion

Adipogenesis of MSCs is a complex and highly regulated process [1]. Understanding the molecular events regulating adipogenesis may lead to an effective treatment of lipid metabolism disorders. The determination of the fate of MSCs requires the selective activation of master regulatory genes that are specific for respective lineages, such as C/EBPα, PPARγ, FABP4, and adiponectin for adipogenesis [1]. Interestingly, recent evidence indicates a role of NRP1 in human adipogenesis of adipose-derived MSCs, with potential implications as a previously unknown mediator of KGF action by regulation of Akt and ERK1/2 phosphorylation [8]. NRP1 is a multiligand single-pass transmembrane receptor that was originally identified as an adhesion molecule and later as a coreceptor for several growth factors. Binding of VEGF-B to VEGFR1 and its coreceptor NRP1 increases endothelial lipid uptake [16]. NRP1 plays an essential role in macrophages in adipose tissue homeostasis [17]. NRP1 is almost not expressed in the primary bone marrow-derived stromal cells (BMSCs), but when BMSCs differentiate towards adipocytic lineage, the NRP1 levels rise [18]. Considering the multiple roles of NRP1 in coreceptor assistance and lipid uptake [16], the propensity of NRP1-expressing myeloid cells to influence adipocyte hypertrophy and fatty liver [17], and its expression in precursors of adipocytes [18], we investigated the implications of NRP1 in the regulation of C3H10T1/2 cells’ adipogenic differentiation.

We found that NRP1 was localized in both the cell membrane and cytoplasm in C3H10T1/2 cells, which is consistent with a growing number of studies that have found that NRP1 is located in multiple subcellular structures. In vascular smooth muscle cells, NRP1 is distributed in both the cell membrane and cytoplasm [9], whereas NRP1 is distributed in both nucleus and cytoplasm in neural crest cells [19]. Interestingly, NRP1 can also be located on the mitochondrial inner membrane and interacts with mitochondrial inner membrane protein in endothelial cells [20]. In addition, NRP1 has been found to be located in the primary cilia of fibroblasts [21]. However, the detailed mechanism by which subcellular localization of NRP1 affects its function remains elusive.

During adipogenic stimulation, insulin binds and activates Akt signaling [22]. Phosphorylated ERK1/2 contributes to the acquisition of DNA-binding activity of C/EBPβ, thereby leading to transcriptional activation of PPARγ and C/EBPα to initiate terminal adipogenic differentiation [23]. We decided to analyze the activation of Akt and ERK1/2 pathway since they are both required not only for mesenchymal cell proliferation but also for the mitotic clonal expansion, which represents the first step of adipogenesis [1]. The function of ERK in adipogenesis seems to be regulated in a timely manner; ERK has to be turned on in the initial proliferative step, while later on, it has to be shut off subsequently to avoid PPARγ phosphorylation for facilitating terminal differentiation [24]. Our data are consistent with this observation, as we demonstrated that ERK is activated only transiently after the addition of adipogenic induction.

Morphological changes are one of the important biological processes that occur in adipogenic differentiation of MSCs. Scaffold proteins have been reported to regulate cell morphology, cell signaling cascades, and gene transcription during differentiation [25]. Our previous studies have identified that scaffold protein JIP4 is a possible NRP1-interacting factor in dental pulp stem cells (DPSCs) (data not published). Recently, it has been shown that JIP4 has a positive influence on the directional differentiation of stem cells from apical papilla (SCAPs) [26]. It has also been demonstrated that JIP4 is a partner protein of one membrane receptor, EGFR [11]. These observations have raised the possibility that NRP1 regulates adipogenic differentiation in C3H10T1/2 cells through its interaction with JIP4. Using co-localization studies, we provided evidence that JIP4 was more intensively recruited to NRP1. Interestingly, the molecular weight of the JIP4 protein detected in the immunoprecipitates was lower than that in the input group. We verified this co-precipitation again in HEK-293T cells and obtained similar results. The reason for this phenomenon is not clear so far. Structurally, NRP1 has two extracellular domains, a1 a2 and b1 b2, in addition to a dimerization domain, transmembrane domain, and short cytoplasmic region (Figure 5A). Since NRP1 lacks kinase activity, there has been a concerted effort to elucidate the mechanisms underlying NRP1 signaling. JIP4 is not the first reported scaffold protein that can directly bind NRP1, but there are few reports on the involvement of JIP4 in adipogenic differentiation. As a scaffold protein, JIP4 plays a pivotal role in regulating the MAPK signaling cascades [10]. In line with other studies [12,13,14], our study suggests that JIP4 has a much broader spectrum of activity. NRP1 and JIP4 may be involved in the cell fate determination of MSCs by stimulating their switch to an adipogenic-like phenotype.

Interestingly, individual knockdown of JIP4 inhibited adipogenic differentiation; however, the inhibitory effect of double knockdown of NRP1 and JIP4 was superior to that of JIP4, but similar to that of NRP1. Hence, JIP4 and NRP1 seem to form a complex, in which NRP1 plays a prominent role. Therefore, it seems reasonable to suggest that the inhibitory effect of NRP1-knockdown overrode the effect of JIP4-knockdown. NRP1 can form complexes with a variety of proteins. For example, NRP1 and the plexin receptor form complexes to mediate secreted Sema3A signal for neuronal axon guidance [5]. GAIP-interacting protein C terminus (GIPC1) is a scaffold protein that also interacts with the NRP1 cytoplasmic region; moreover, NRP1 induces cancer cell proliferation by forming a complex that contains GIPC1 [27]. These reports, together with our results, indicate that NRP1 interacts with JIP4. However, this point remains to be clarified.

NRP1 has a very clear and definite domain composition. Disruption of either intracellular or extracellular domains affects NRP1 function. NRP1 splice variants, which are generated by skipping exons 4 and 5, result in defects in N-linked glycosylation at asparagine (N) positions N150 and N261, thereby exhibiting increased endocytosis/recycling activity with decreased levels of degradation [28]. Here, we generated an NRP1 S612A mutant, which defect the modification of the addition of GAG to a single conserved serine residue (Ser612). Surprisingly, overexpression of the NRP1 S612A mutant in C3H10T1/2 cells led to enhanced adipogenic differentiation, with markedly increased levels of phosphorylated Akt and ERK. This effect was not due to an increase in the total level of Akt and ERK. It has also been reported that NRP1 S612A cells showed a significant increase in cell invasion in a three-dimensional (3D) matrix via upregulating p130Cas phosphorylation [29]. In addition, it has been suggested that glycosylation of programmed death ligand-1 (PD-L1) antagonizes glycogen synthase kinase 3β (GSK3β) binding to PD-L1, while only non-glycosylated PD-L1 forms a complex with GSK3β [30]. Similar to that study, we found that NRP1 without glycosylation was able to bind to JIP4 (Figure S1). We have not ruled out the possibility that GAG modification of NRP1 induces conformational changes in the binding surface between NRP1 and its cooperation protein. It is possible that glycosylation at Ser612, a post-translational modification, can maintain the homeostasis of NRP1 and inhibit NRP1 overactivation. The hypotheses, however, clearly require further studies for their validation. An increasing body of evidence has shown that glycosylation usually alters protein localization, stability, and function [31]. It has been found that the interaction between the Sema3A C-terminal polybasic region and GAG units can potentiate the Sema3A signaling pathway [32]. A small peptoid molecule could interfere with the interaction between the Sema3A C-terminal region and GAGs by displacing the Sema3A C-terminal domain from the GAGs or by binding directly to them [32]. This intriguing report raises the possibility that future studies should develop protein–GAG interaction inhibitors of therapeutic interest.

**Figure 6:**
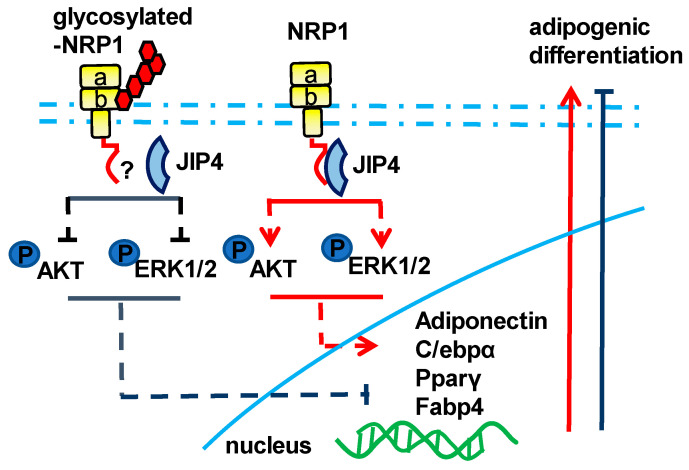
Schematic of the NRP1–JIP4–Akt/ERK signaling pathway promoting adipogenic differentiation of C3H10T1/2 cells.

In summary, our study contributes to clarifying the molecular basis of adipogenesis, pointing out the role of NRP1 in conjunction with JIP4 as a positive mediator of the Akt and ERK pathways in the adipogenic differentiation of C3H10T1/2 cells. In addition, the glycosylated modification of NRP1 at Ser612 plays a brake or decelerator role in promoting adipogenesis (Figure 6). Our results may provide a basis for the development of new reagents and drugs and for identifying the role of post-translation modifications of key proteins in the adipogenic differentiation of stem cells.

## 4. Materials and Methods

### 4.1. Cell Culture and Differentiation

C3H10T1/2 cells, a mouse MSC line, were obtained from the Riken Cell Bank (RCB0247, Tsukuba, Japan). The cells were cultured in 1:1 Ham’s F12:DMEM complete medium containing 10% fetal bovine serum (FBS) (Gibco, Carlsbad, CA, USA) and 1% penicillin/streptomycin (Gibco), and were incubated in a 5% CO_2_ atmosphere at 37 °C.

For induction of adipogenic differentiation, confluent C3H10T1/2 cells were cultured with 10 µg/mL insulin (Sigma, St. Louis, MO, USA), 1 µM dexamethasone (Sigma), and 0.5 mM 3-isobutyl-1-methylxanthine (IBMX) (Sigma). After two days, the cells were maintained in a complete medium supplemented with 10 µg/mL insulin for the remainder of the study.

### 4.2. Oil Red O Staining and Quantification

To prepare the oil red O stock solution, 500 mg of oil red O powder (Nacalai Tesque, Kyoto, Japan) was dissolved in 100 mL of 99% isopropanol. Theoil red O working solution was prepared just before use by diluting 60 mL of the stock solution with 40 mL of distilled water. To measure lipid accumulation, the cells were washed with phosphate-buffered saline (PBS), fixed with 10% formalin for 15 min, rinsed with 60% isopropanol, and stained with oil red O working solution for 15 min at 37 °C, followed by washing with PBS. Brightfield images were taken with identical exposures on a Nikon DS-L2 camera. Oil red O was extracted with 99% isopropanol and the absorbance was measured at 490 nm using Microplate Reader SH-1000Lab (Corona, Ibaragi, Japan). Empty wells stained with oil red O working solution were used as background, and absorbance was subtracted from each sample for quantification.

### 4.3. SDS-PAGE and Western Blotting

Total protein lysates were harvested in lysate buffer (1 mM dithiothreitol, 1 mM phenylmethylsulfonyl fluoride, 1 μg/mL leupeptin, 2 μg/mL aprotinin, 5 mM EGTA) and sonicated on ice using a supersonic machine. Protein concentrations were determined with the Protein Assay Reagent (Bio-Rad, Hercules, CA, USA). Equal amounts of protein were separated using 10% SDS-PAGE gels and transferred to PVDF membranes (Millipore, Billerica, MA, USA). The following primary antibodies were used at a dilution of 1:1000 overnight at 4 °C: NRP1 (Abcam, Cambridge, UK), JIP4 [Cell Signaling Technology (CST), Inc., Danvers, MA, USA], phosphorylated Akt (CST), Akt (CST), phosphorylated ERK (CST), ERK (CST), and GAPDH (CST). Signal was detected with horseradish peroxidase–conjugated secondary antibodies (diluted at 1:2000) and Immobilon Forte Western HRP substrate detection substrate (Millipore) using a Bio-Rad Chemidoc imaging system. The proteins were normalized to GAPDH, and the intensity of each band was quantified using ImageJ software (National Institutes of Health, Bethesda, MD, USA).

### 4.4. Immunocytochemistry

C3H10T1/2 cells were fixed with 4% paraformaldehyde for 15 min and then permeabilized with 0.1% Triton X-100 in PBS for 3 min on ice. After blocking with 5% bovine serum albumin (BSA) in PBS for 2 h, the cells were incubated with corresponding antibodies. For localization of NRP1 and β-actin, the cells were incubated with NRP1 antibody (Abcam; 1:250) overnight at 4 °C, and then incubated with Alexa Fluor 594–conjugated secondary antibodies (Invitrogen, Carlsbad, CA, USA) (1:300), followed by staining with ActinGreen™ 488 ReadyProbes™ Reagent (Invitrogen). The stained cells were counterstained with DAPI and were observed using a confocal laser scanning microscope LSM780 (Carl Zeiss, Oberkochen, Germany) at Central Research Laboratory, Okayama University Medical School. The procedure of colocalization of NRP1 and JIP4 with immunocytochemistry was shown in Supplementary Materials and Methods.

### 4.5. Reverse-Transcription and Quantitative Real-Time PCR (qPCR)

RNA was isolated from C3H10T1/2 cells using Trizol reagent (Molecular Research Center, Cincinnati, OH, USA) in accordance with the instructions of the manufacturer. cDNA was generated from 1 μg of mRNA using PrimeScript™ RT Master Mix (Takara, Kyoto, Japan). Analysis of gene expression was performed using Luna^®^ Universal qPCR Master Mix (New England Biolabs, Ipswich, MA, USA) in a LightCycler System (Roche Diagnostics, Mannheim, Germany). The relative number of mRNAs was determined through the comparative threshold cycle (ΔΔCT) method and was normalized to the levels of β-actin mRNA. Primer sequences are listed in Supplementary Table S1.

### 4.6. NRP1 shRNA Knockdown

Based on the results of bioinformatics, we selected two short-hairpin RNA (shRNA) molecules targeting mouse NRP1 sequence (sh1: 5′-CCGG GGA AAT AAA GCC ATT ATC TTT CTC GAG AAA GAT AAT GGC TTT ATT TCC TTT TTG-3′; sh2: 5′-CCGG GAT GAT ATC AGT ATT AAC AAC CTC GAG GTT GTT AAT ACT GAT ATC ATC TTT TTG-3′). The lentivirus vector pLKO.1-puro used in this experiment was produced and kindly provided by Dr. Takarada (Okayama University). Briefly, two single-stranded DNA molecules were designed and synthesized. Then, double-stranded DNA, formed by annealing the single-strands, was connected to the linearized vector, and was transformed into *E. coli* DH5α competent cells (Takara). The plasmid was extracted and sequenced, and the lentivirus was packed with HEK 293T cells.

C3H10T1/2 cells were transduced with lenti-sh1, lenti-sh2, or empty vector (referred to as lenti-sh-cont). Following transduction, the cells were selected with 5 μg/mL puromycin. Then, stable single-cell clones were selected and verified by qPCR and Western blotting. The clones sh-cont, sh1-1, sh1-2, sh2-1, and sh2-2 were finally selected for subsequent experiments.

### 4.7. JIP4 siRNA Transfection

C3H10T1/2 cells were transfected with Ambion Silencer Select siRNA targeted to mouse JIP4 (Thermo Fisher Scientific, Waltham, MA, USA), or negative control siRNA (Invitrogen) at a final concentration of 50 nM, using 2 μg/mL polyethylenimine (PEI) (Invitrogen) in an antibiotic-free medium. All experiments were performed 48 h after transfection, and the efficiency of transfection was confirmed using qPCR and Western blotting.

### 4.8. Lentiviral and Retroviral Vector Production and Infection

The mouse NRP1 wild type with Flag (NRP1 WT), NRP1 S612A mutant with Flag (NRP1 S612A), and control plasmid (cont) were designed, synthesized, and cloned into the GV218 vector by the GeneChem Corporation (Shanghai, China). Pseudotyped lentiviral vectors were produced in HEK293T cells transiently co-transfected with 10 μg of the corresponding lentiviral vector plasmid, 6 μg of the packaging plasmid pSPAX2 (Addgene, Watertown, MA, USA), and 6 μg of the VSV g envelope protein plasmid pMD2G (Addgene) using Lipofectamine Plus reagent, in accordance with the manufacturer’s instructions (Invitrogen). The cells were infected in the presence of 4 μg/mL polybrene (Sigma-Aldrich) and selected with 10 μg/mL puromycin (Sigma-Aldrich).

### 4.9. Co-Immunoprecipitation (Co-IP)

C3H10T1/2 cells cultured in 100 mm plastic dishes were washed twice with PBS, scraped into PBS, pelleted at 3000× *g*, and resuspended in 1 mL of lysis buffer (5 M NaCl, 10% NP-40, and 1 M Tris–HCl, pH 8.0). The lysate was incubated overnight at 4 °C in rotation with 40 μL anti-Flag M2 affinity gel (Sigma). On the next day, the complex was washed five times with lysis buffer and incubated for 30 min with 100 μg/mL of Flag Peptide (Sigma) at 4 °C in rotation. The supernatant was separated in a fresh tube and 20 μL of electrophoresis sample buffer was added, boiled for 5 min, and examined via Western blotting.

### 4.10. Statistical Analysis

All data are presented as the mean ± standard deviation (SD). Data were analyzed using either a one-way analysis of variance (ANOVA), followed by a Tukey’s post hoc test for comparison of multiple groups, or an independent Student’s *t*-test for comparison of two groups. Asterisks indicate statistically significant differences (*, *p* < 0.05). All of the statistical analyses were performed using GraphPad Prism 8 for Windows (GraphPad Software, Inc., San Diego, CA, USA). The experiments were repeated at least three times, and representative images are shown in the figures.

## Data Availability

The data presented in this study are available upon request from the corresponding author.

## Supplementary Materials

The supporting information can be downloaded at: https://www.mdpi.com/article/10.3390/ijms24087394/s1.

## Abbreviations

MSCs	Mesenchymal stem cells
NRP1	Neuropilin 1
GAG	Glycosaminoglycan
JIP4	C-Jun N-terminal kinase (JNK)–interacting protein 4
MAPK	Mitogen-activated protein kinase
ERK	Ras-extracellular signal-regulated kinase
JNK	C-Jun N-terminal kinase
Co-IP	Co-immunoprecipitation
C/EBPα	CCAAT/enhancer binding protein α
PPARγ	Peroxisome proliferator-activated receptor γ
FABP4	Fatty acid-binding protein 4
