# Supplementary material for: Neuropilin 1 (NRP1) Positively Regulates Adipogenic Differentiation in C3H10T1/2 Cells
Source: Int J Mol Sci. 2023 Apr 17;24(8):7394. doi: 10.3390/ijms24087394 (PMC10138427; doi:10.3390/ijms24087394)
Supplement: Supplementary file 1 [file ijms-24-07394-s001.zip › ijms-2316811-supplementary.pdf]

Neuropilin 1 (NRP1) positively regulates adipogenic differentiation in C3H10T1/2 cells

Yaqiong Yu 1,Yoko Uchida-Fukuhara 2, Yao Weng 2, Yuhan He 1, Mika Ikegame 2, Ziyi Wang 3 , Kaya Yoshida 4, Hirohiko Okamura 2*, Lihong Qiu 1*

Supplementary Materials and methods

Colocalization of NRP1 and JIP4 with immunocytochemistry

After differentiation induction into adipocytes for three days, ov-NRP1 WT cells and ov-NRP1 S612A cells were fixed and then permeabilized As described in 4.4. Immunocytochemistry in Materials and Methods. For localization of NRP1 and JIP4, the cells were incubated with JIP4 antibody (CST; 1:250) overnight at 4°C, and then incubated with Alexa Fluor 594–conjugated secondary antibodies (Invitrogen, Carlsbad, CA, USA) (1:300), followed by Stained with DAPI and were observed using a confocal laser scanning microscope LSM780 (Carl Zeiss, Oberkochen, Germany) at Central Research Laboratory, Okayama University Medical School. Quantitative colocalization analysis was performed on raw images using the “Colocalization Threshold” function in ImageJ. Pearson's correlation coefficient (Pearson's r) was applied to estimate the colocalization level using all pixels above the automatically determined threshold.

Supplementary Table S1

Real time-PCR primer sequences

Gene	Forward	Reverse
Nrp1	5’-AGAGAATCATAATCAACTTCAACCC-3’	5’-CATAGCGGATGGAAAACCCTG-3’
Spag9	5’-ACATGACAAAGTCTGGTGTGGCTA-3’	5’-AAGCTGCCGTACTTGGCTCTC-3’
Pparg	5’-GGAGCCTAAGTTTGAGTTTGCTGTG-3’	5’-TGCAGCAGGTTGTCTTGGATG-3’
Cebpa	5’-TTGAAGCACAATCGATCCATCC-3’	5’-GCACACTGCCATTGCACAAG-3’
Fabp4	5’-TGGGAACCTGGAAGCTTGTCTC-3’	5’-GAATTCCACGCCCAGTTTGA-3’
Adipoq	5’-TTCTGTCTGTACGATTGTCAGTGGA-3’	5’-GGCATGACTGGGCAGGATTA-3’

Neuropilin 1 (NRP1) positively regulates adipogenic differentiation in C3H10T1/2 cells

Yaqiong Yu 1,Yoko Uchida-Fukuhara 2, Yao Weng 2, Yuhan He 1, Mika Ikegame 2, Ziyi Wang 3 , Kaya Yoshida 4, Hirohiko Okamura 2*, Lihong Qiu 1*

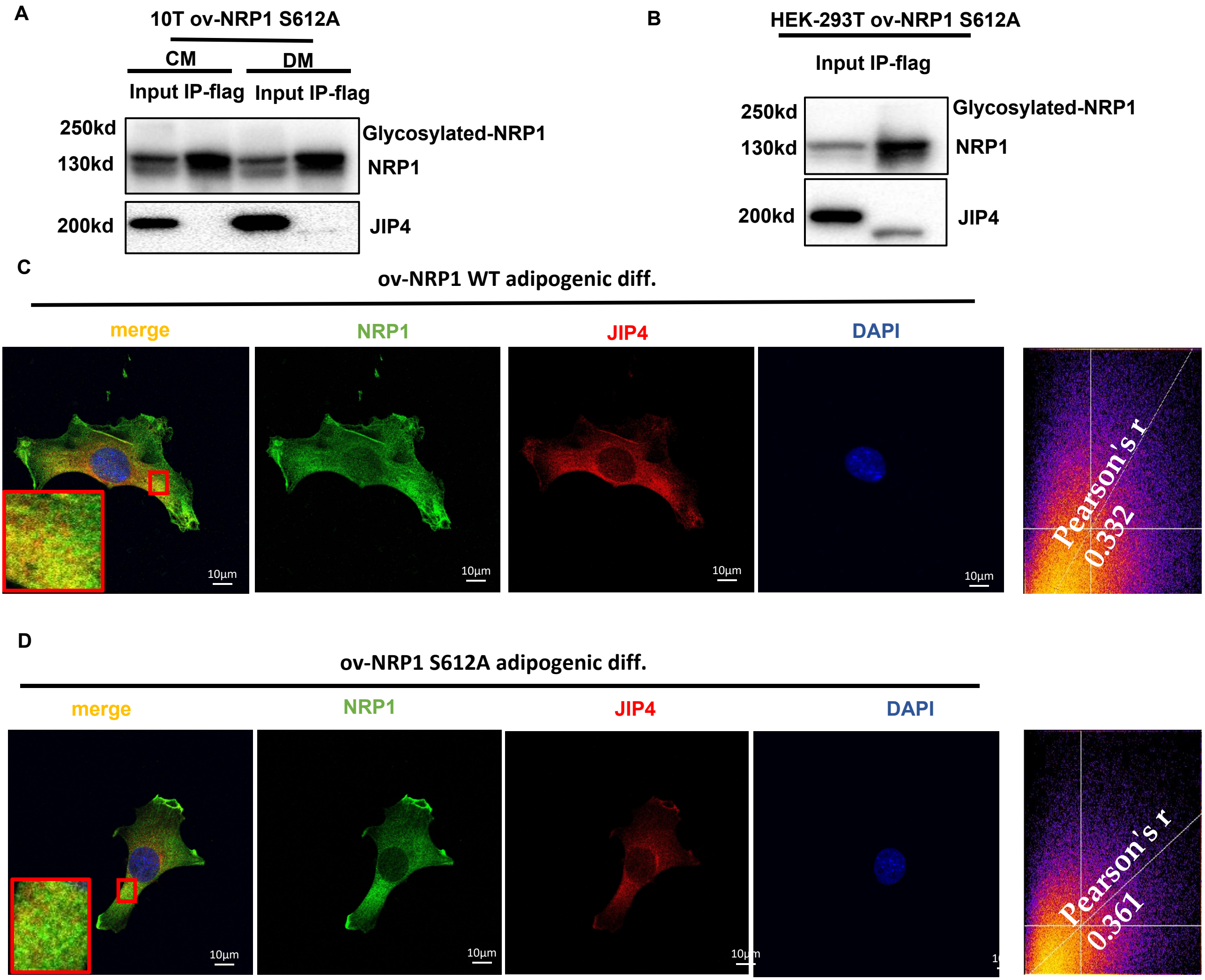

Figure S1. Nonmodifiable NRP1 mutant (S612A) binds to JIP4 in adipogenic differentiation. Samples from ov-NRP1 S612A from C3H10T1/2 cells (A) or HEK 293T cells (B) were immunoprecipitated with anti-Flag antibody–conjugated agarose and subjected to Western blot analysis. Whole-cell lysate from the cells was used as positive control (input). The blotted membranes were incubated with anti-NRP1 antibody (upper panel) or anti-JIP4 antibody (lower panel). (C) ov-NRP1 WT and (D) ov-NRP1 S612A C3H10T1/2 cells were cultured in an adipogenic medium for three days. Immunostaining was performed using an anti-JIP4 antibody (red) (Scale bar = 10 μm). Representative scatter plots are shown. The white horizontal and vertical lines indicate the automatically determined thresholds. The Pearson's r was applied to estimate the colocalization level using all pixels that above the automatically determined threshold.The images are representative of at least three independent experiments. Immunoprecipitation (IP); control medium (CM); differentation medium (DM).
